# A Critical Review of White Matter Changes in Huntington’s Disease

**DOI:** 10.1002/mds.28109

**Published:** 2020-06-15

**Authors:** Chiara Casella, Ilona Lipp, Anne Rosser, Derek K Jones, Claudia Metzler‐Baddeley

**Affiliations:** ^1^ Cardiff University Brain Research Imaging Centre School of Psychology, Cardiff University Cardiff United Kingdom; ^2^ School of Biosciences Cardiff University Cardiff United Kingdom; ^3^ Department of Neurophysics Max Planck Institute for Human Cognitive and Brain Sciences Leipzig Germany; ^4^ Mary MacKillop Institute for Health Research Australian Catholic University Melbourne Victoria Australia

**Keywords:** Huntington’s disease, MRI, myelin, oligodendrocytes, white matter microstructure

## Abstract

Huntington’s disease is a genetic neurodegenerative disorder. White matter alterations have recently been identified as a relevant pathophysiological feature of Huntington’s disease, but their etiology and role in disease pathogenesis and progression remain unclear. Increasing evidence suggests that white matter changes in this disorder are attributed to alterations in myelin‐associated biological processes. This review first discusses evidence from neurochemical studies lending support to the demyelination hypothesis of Huntington’s disease, demonstrating aberrant myelination and changes in oligodendrocytes in the Huntington’s brain. Next, evidence from neuroimaging studies is reviewed, the limitations of the described methodologies are discussed, and suggested interpretations of findings from published studies are challenged. Although our understanding of Huntington’s associated pathological changes in the brain will increasingly rely on neuroimaging techniques, the shortcomings of these methodologies must not be forgotten. Advances in magnetic resonance imaging techniques and tissue modeling will enable a better in vivo, longitudinal characterization of the biological properties of white matter microstructure. This in turn will facilitate identification of disease‐related biomarkers and the specification of outcome measures in clinical trials. © 2020 The Authors. *Movement Disorders* published by Wiley Periodicals, Inc. on behalf of International Parkinson and Movement Disorder Society.

Huntington’s disease (HD) is a genetic neurodegenerative disorder that leads to debilitating cognitive, psychiatric, and motor symptoms. The mutation accountable for HD is an expansion of the cytosine‐adenine‐guanine (CAG) repeat within the huntingtin (*HTT*) gene. A clinical diagnosis of symptomatic HD requires the onset of motor abnormalities such as chorea, motor impersistence (ie, the inability to sustain simple voluntary movements), along with the presence of a family history of the disease.^1^ Currently, HD cannot be cured, and a research priority is to increase the understanding of its pathogenesis and to provide biomarkers for evaluating the efficacy of targeted therapies.

Although HD pathology is tightly associated with the degeneration of striatal gray matter (GM),^2^ during the past years HD research has identified white matter (WM) changes as relevant pathophysiological features of HD.^1^, ^3^, ^4^, ^5^, ^6^, ^7^, ^8^, ^9^, ^10^ However, despite a subcortical WM volume loss of 29% to 34% already having been reported in postmortem HD brains more than 20 years ago,^11^ the etiology of WM degeneration and its role in disease pathogenesis and progression remain unclear.

Although some work suggests that WM damage in HD is secondary to the loss of GM volume in the form of Wallerian degeneration,^11^ there is evidence suggesting that WM aberrations are a feature of HD that occurs independent of neuronal cell loss.^1^, ^5^, ^8^, ^12^, ^13^, ^14^, ^15^, ^16^, ^17^ Accordingly, WM changes are present very early in the disease course, even in children at risk for HD,^18^ and in premanifest individuals who are more than 15 years away from symptom onset.^4^, ^17^, ^19^ Notably, WM is composed of axons as well as nonneuronal glia cells, such as myelin‐producing oligodendrocytes, and it is unclear whether axons, myelin, or both are predominantly responsible for the WM loss.^7^

An increasing body of research suggests that WM in HD is subject to alterations in myelin‐associated biological processes at the cellular and molecular levels.^13^, ^15^, ^20^, ^21^, ^22^, ^23^, ^24^ Myelin is an axon‐wrapping, multilayered sheath and is produced by oligodendrocytes. Axon myelination is vital during brain development and critical for healthy brain function as it plays a fundamental role in the regulation of efficiency and speed of action potential propagation during synaptic transmission.^25^, ^26^ Dysfunction in oligodendrocytes and myelin can slow down or stop otherwise fast axonal transport, which in turn can result in synaptic loss and eventually axonal degeneration.^27^ Furthermore, as myelination patterns across the brain enable the temporal synchronization of information processing, local demyelination can disrupt the performance of functional networks.^28^

The demyelination hypothesis of HD^4^ suggests that mutant huntingtin (the protein produced by the huntingtin gene) leads to premature myelin breakdown in HD. A dysfunction of oligodendrocytes in turn might impair the repair of demyelinated axons, leading to chronic demyelination. Alternatively, as oligodendrocytes are the major iron‐containing cells of the central nervous system, it might be that homeostatic increases in these cells, related to their role in remyelinating axons, cause significant increases in ferritin iron content. High ferritin iron is proposed to have toxic effects and could further contribute to impairments in WM and myelination.^4^, ^29^, ^30^

The objective of the present work was to systematically review the impact of HD pathology on WM microstructure and to evaluate evidence on the demyelination hypothesis,^4^ which was initially proposed 10 years ago.

To look for relevant articles, we performed a search in PUBMED and GoogleScholar.com using the following keywords: Huntington’s disease, white matter, white matter microstructure, myelin, myelination, diffusion tensor imaging, magnetic resonance imaging, mouse models of Huntington’s disease, premanifest, presymptomatic, symptomatic, oligodendrocytes, iron. The articles identified were short listed based on their titles and abstracts. The reference sections of relevant articles were also used to search for additional relevant articles. The criteria for inclusion in this review were the following: studies describing WM differences and changes in HD mutation carriers either at the premanifest or symptomatic stage and studies on animal models of HD investigating molecular or cellular processes linked to WM microstructure or assessing HD‐related changes in brain structure with magnetic resonance imaging (MRI). A total of 103 studies, published between 1973 and 2020, were reviewed; 69 of these described research involving patients with HD.

## WM Impairment in HD: Secondary to, or Independent of, Neuronal Degeneration?

WM changes have been reported both in animal models and in HD human carriers by histopathological postmortem studies^11^, ^21^, ^22^, ^31^ and MRI studies.^4^, ^9^, ^17^, ^22^, ^32^, ^33^, ^34^ These have shown widespread WM volume loss^1^, ^5^, ^6^, ^17^, ^19^, ^33^, ^34^, ^35^ and WM alterations at the microstructural and molecular levels.^4^, ^8^, ^10^, ^14^, ^21^, ^22^, ^33^, ^36^, ^37^, ^38^

The earliest WM alterations are seen in HD human carriers years before the onset of signs and symptoms of manifest HD. Prominent areas of damage include the striatum, the corpus callosum (CC), and posterior WM tracts.^12^, ^34^, ^36^, ^39^, ^40^ Furthermore, the severity of WM changes has been shown to correlate with predicted time to symptom onset in premanifest patients,^1^, ^6^, ^38^ with measures of motor dysfunction^33^ and with cognitive deficits.^33^, ^41^

### Evidence From Neurochemical Studies

This section reviews evidence for molecular and neurochemical changes in oligodendrocyte lineage cells and myelin sheaths in HD. These changes have been reported in some human subjects and also in studies of animal models of HD.

The phenotype exhibited by each animal model of HD needs to be considered in the context of the different approaches used to generate the respective model.^42^ Key distinguishing factors are the genetic approach and transgene construct used, for example, the use of full‐length or only a fragment of mutant huntingtin (*HTT*), the length of the CAG repeat incorporated into the genome, the expression of the HD mutation from a transgene versus knock‐in of the mutation into the endogenous HTT locus. In general, because knock‐in models carry the mutation in its appropriate genomic and protein contexts, they are considered as more accurate genetic models of the human condition compared with transgenic models.^43^

Importantly, findings from animal models of HD need to be considered while keeping in mind the inherent differences between these animal and human models. Specifically, the equivalent and divergent features of the brain for different species need to be understood to ensure that data extrapolation is performed rationally. For example, when looking at rodent brains, the most obvious difference is that these are tiny (~0.4 g in mice, ~2.0 g in rats), lissencephalic (do not have sulci or gyri), and have little WM. On the other hand, the human brain is much larger (approximately 1300 g) and has a readily evident lobular organization, prominent sulci and gyri, and extensive WM (about 40% of the brain).^44^

#### Abnormalities of Oligodendrocyte Lineage Cells in HD

An increase in oligodendroglia differentiation in neural progenitor cells was observed during postnatal development in transgenic HD rats.^45^ Similarly, enhanced proliferation of oligodendrocyte precursor cells was found in adult HdhQ250 mice.^22^ In addition, Simmons and colleagues^23^ reported increased immunostaining for ferritin, an iron storage protein that is mostly found in oligodendrocytes, in the striatum, cortex, and hippocampus of the R6/2 transgenic mouse model of HD and detectable before any behavioral abnormalities could be observed. Human postmortem histopathological studies have also demonstrated an increase in the density of oligodendrocytes in the striatum of patients with HD compared with healthy controls, years before striatal atrophy or a loss of neurons occurs.^15^, ^20^ The increased number of oligodendrocytes observed suggests the presence of a homeostatic myelin repair mechanism, aiming to compensate deficits in myelination that seem to occur in HD.

In contrast, a study using carbon‐14 dating approaches indicated that oligodendrocyte lineage cells are the most depleted cell types among all nonneuronal cells in the symptomatic HD brain.^46^ Furthermore, a dramatically lower number of mature oligodendrocytes during the postnatal myelination period has been shown in the HdhQ250 knock‐in mouse model of HD.^22^ This evidence suggests that HD might be associated with a lack of oligodendrocytes, which cannot repair demyelinated axons. Accordingly, silencing oligodendroglia‐specific mutant‐HTT (*mHTT*) expression in BACHD mice rescues deficits in the thickness and compactness of myelin sheaths that otherwise occur in these mice,^3^ and expressing *mHTT* selectively in the oligodendrocytes of transgenic mice induces impairments in myelination.^21^ Overall, the present findings suggest that there is a relationship between the HD mutation and oligodendrocyte dysfunction.

To summarize, although increased numbers of oligodendrocytes have been observed, their dysfunctionality may lead to unsuccessful myelination. It is also possible that the observed increased levels of oligodendrocytes are helpful at first but eventually lead to toxicity because of increased iron levels. Both explanations fit within the demyelination hypothesis^4^ as they implicate an increasingly unsuccessful compensation for the disease‐related myelin loss.

#### Myelin Changes in HD

Reports from human postmortem studies have demonstrated a striking breakdown of myelin in the HD brain.^47^ In addition, some studies on animal models have indirectly demonstrated impairments in developmental myelination in HD: these have shown that the expression of a mutant huntingtin transgene in cells and in R6/2 transgenic mice leads to reduced activity in the cholesterol biosynthesis pathway that in turn results in lower levels of newly synthesized cholesterol and its intermediates,^48^, ^49^ which are essential for the synthesis of myelin.^50^

Electron microscopy investigations have reported thinner myelin sheaths, as reflected by higher g‐ratios (the ratio of the inner axonal diameter to the outer diameter), in transgenic BACHD rats and in the HdhQ250 knock‐in mouse model.^22^, ^24^ HD myelin changes might either represent a breakdown of myelin into fragments because of toxicity or thinner myelin sheaths because of a developmental dysfunction in myelination mechanisms. Consistent with the latter, alterations in myelin sheaths are paralleled by the reduced expression of myelin‐related genes such as myelin basic protein (*MBP*) and myelin oligodendrocyte glycoprotein (*MOG*) in transgenic R6/2 and HdhQ250 knock‐in mice.^22^, ^51^, ^52^ Reduced levels of *MBP* and *MOG* in brain regions known to be affected by HD throughout the critical postnatal myelination stage, and significantly fewer myelinated axons, have been reported in knock‐in HdhQ250 mice.^22^ The reduction in myelin proteins may be attributed to a decreased expression of myelin regulatory factor (*MRF*), a transcription factor that controls the expression of myelin‐related proteins.^22^ Moreover, abnormalities in myelin sheaths and myelin‐related gene transcripts in YAC128 transgenic mice are evident well before any striatal neuronal loss can be detected.^24^ These findings imply that a dysregulation of the temporal profile of myelination might underlie WM abnormalities and that disordered myelination during the postnatal period might constitute an important early pathogenic event in HD.^22^

### Evidence From Imaging Studies

Neuroimaging techniques enable the assessment of brain structure and function in vivo and allow the understanding of disease pathology over time. However, as much of our understanding of HD pathology will increasingly rely on advanced neuroimaging techniques, it is important to bare in mind the limitations of these approaches. Table 1 provides a summary of MRI results observed in patients with HD in studies discussed in this review. Interpretations proposed by the respective authors and alternative explanations found in the literature for such changes are reported. Table 2 summarizes MRI studies reporting WM changes in HD animal models.

**Table 1:** MRI changes observed in the reviewed in vivo studies in patients with HD: interpretations proposed by the respective authors and alternative explanations found in the literature for such changes

Reported Change	Proposed Interpretation	Possible Alternative Interpretation
Reduced WM volume	Decreased number of axons attributed to Wallerian degeneration^50^, ^51^, ^52^, ^53^, ^54^	Decrease in axon myelination^5^, ^6^
Reduced axial diffusivity	Axonal degeneration^2^	Inflammation, nonuniform axonal oedema, beads, varicosities parallel to the axon segments, microglia/macrophage activation^100^
Increased radial diffusivity	Demyelination^2^, ^9^, ^18^, ^60^, ^69^, ^70^	Less coherent alignment of fibers, more crossing fibers from other bundles, lower density or less myelination of the fibers, or a combination of any or all these factors^101^
Reductions in the neurite density index	Decrease in axonal density^76^	Reduced MRI signal because of demyelination^102^
Reductions in MPF	Demyelination^59^	Changes in cells and water content attributed to inflammation^79^, ^83^
Shortened T2	Increased ferritin levels^86^, ^89^	Remyelination^103^

MPF, macromolecular proton fraction; MRI, magnetic resonance imaging; WM, white matter.

**Table 2:** Summary of reviewed MRI studies of HD animal models

Species/Model	MRI Technique (In Vivo/Ex Vivo)	Findings	Neurochemical Validation	Reference
R6/2 mice	DT‐MRI (ex vivo)	FA reductions in genu and splenium of the corpus callosum.	Yes	49
YAC128 mice	Structural MRI (ex vivo)	Progressive loss of WM volume. Corpus callosum, anterior commissure, and fimbria are among the most discriminatory areas in genotype separation.	No	99
YAC128 mice	DT‐MRI (in vivo)	FA reductions in the anterior commissure, corpus callosum, internal capsule, and external capsule, from 1.5 months of age; in the cingulum and cerebral peduncle from 3 months of age.	Yes	23
BACHD rats	DT‐MRI (in vivo)	FA reductions in the anterior corpus callosum, the cingulum, and the external capsule at 12 months of age.	Yes	23
TgHD rats	DT‐MRI and PET (in vivo)	Increased MD in HD rats at 12 months of age, compared with earlier time points; this parameter remained constant in WT animals. Age‐related RD decreases at 6 months of age in HD animals but only at 12 months in WT animals.	Yes	94
TgHD rats	Diffusion kurtosis imaging (in vivo)	Neuronal development in HD rat pups occurs differently compared with controls: higher MD values at P15 but lower MD and AD values at P30 in external capsule.	Yes	48
rHD1 rhesus monkeys	DT‐MRI (in vivo)	Widespread WM changes in FA, MD, and RD. HD monkeys reached the maximal FA value earlier (22.7 ± 4.8 months) compared with controls (47.8 ± 11.7), revealing an arrest of WM maturation in the HD group; across ages, HD monkeys had significantly lower maximal FA values in all areas investigated. Significantly higher minimum RD values of HD monkeys in the striatal bundle.	No	31

WM disturbance appears to be an early pathogenic event. An altered developmental trajectory of WM is suggested by asymmetric age‐related changes of MRI metrics between HD models and wild types.

AD, axial diffusivity; DT‐MRI, diffusion‐weighted magnetic resonance imaging; FA, fractional anisotropy; HD, Huntington’s disease; MD, mean diffusivity; MRI, magnetic resonance imaging; PET, positron emission tomography; RD, radial diffusivity; WM, white matter.

Structural neuroimaging studies in animal models of HD and in HD carriers have shown that WM atrophy can be found across several WM areas, including the CC, the anterior commissure, internal and external capsules, and the cingulum. Furthermore, they suggest that these WM changes happen very early in the disease course.^6^, ^12^, ^17^, ^19^, ^53^ Importantly, deficits in brain growth and WM changes are already found in children at risk for HD,^18^, ^54^ further pointing to a neurodevelopmental effect of *mHTT*.

Some studies have suggested that WM is more affected than GM in the HD brain. For example, Tabrizi and colleagues^35^ showed that the rate of change of WM volume over 24 months was greater than that of GM in patients with premanifest and early HD.^35^ Specifically, individuals carrying the *mHTT* gene who were far away from clinical diagnosis showed WM loss only around the striatum and within the CC and posterior WM tracts, whereas those close to clinical diagnosis and patients with symptomatic HD showed extensive WM loss across the whole brain. Loss of WM volume during this time period ranged between 2% in HD carriers more than 10 years away from disease onset and 4% in patients with early HD. Effect sizes for atrophy rates between early HD participants and healthy controls were larger in WM (1.70, 1.40–2.08) than in GM. In addition, a 2‐year longitudinal study found that, when controlling for normal age‐related variations, brain atrophy of premanifest patients was more pronounced in WM than in striatal GM.^19^ Similarly, in another patient cohort, WM volume was drastically reduced in premanifest participants when compared with controls (35.3 ± 2.5 mL vs. 37.7 ± 2.2 mL), whereas no significant differences were observed for GM volumes.^6^

The findings summarized previously suggest that there might be a dissociation between neurodegenerative processes that happen in GM and WM aberrations in HD. Furthermore, the temporal pattern of reported WM changes suggests that WM impairment in HD unlikely reflects solely a secondary result of neuronal cell death in GM and may instead represent an independent factor of HD pathology. Evidence for a link between GM and WM changes comes from studies that show significant atrophy of the cortical mantle of patients with HD both cross‐sectionally^55^, ^56^, ^57^, ^58^ and longitudinally,^53^ suggesting that WM volume loss may be a consequence of the withdrawal of axons projecting from cortical neurons.

These studies have relied on measures of WM volumes. However, WM volume loss as quantified using structural MRI is a rather unspecific marker of disease stage and progression as it is not sensitive to changes in microstructure. This makes it hard to capture differential effects across the various stages of the disease.^59^ As such, reductions in WM volume observed in structural neuroimaging studies can be the consequence of several factors, including a decrease in the number of axons because of Wallerian degeneration,^53^, ^55^, ^56^, ^57^, ^58^ a decrease in axon myelination,^6^, ^60^ or a combination of both.

New MRI methods allow us to move beyond traditional macrostructural volumetric methods and provide more in‐depth information about tissue integrity and organization at the microstructural and biochemical levels.

To date, most neuroimaging studies of WM microstructure have used diffusion tensor MRI (DT‐MRI)^61^ to quantify tissue properties. This technique characterizes the 3‐dimensional diffusion of water as a function of spatial location, and it is based on the differential diffusion of water molecules depending on tissue type and architecture.^62^ For example, the molecular diffusion rate (mean diffusivity), the directional preference of diffusion (fractional anisotropy [FA]), the diffusion rate along the main axis of diffusion (axial diffusivity [AD]), and the rate of diffusion in the transverse direction (radial diffusivity [RD]) can be inferred.

Some evidence from DT‐MRI studies suggests that WM aberrations in HD are a consequence of Wallerian axonal degeneration. For example, it has been shown that WM changes correlate with reductions in cerebral GM density.^9^, ^12^ Similarly, a DT‐MRI study suggested that WM changes in HD are a consequence of axonal injury rather than demyelinating mechanisms based on the observation of greater changes in AD, as compared to RD changes, in the brains of patients with HD.^2^

On the other hand, there are several DT‐MRI studies that have suggested a role for myelin in HD pathology.^4^, ^9^, ^12^, ^14^, ^63^, ^64^ These studies parallel findings from postmortem, histopathological studies in patients with HD.^47^ First, insufficient myelination was suggested to be present in children at risk of HD, as they showed increased RD in the external capsule. Specifically, the authors hypothesized that this increase may reflect an impairment in myelin integrity because of a dysfunction in the trophic support mechanisms usually carried out by normal *HTT*. This in turn is suggested to affect myelin integrity by hindering the production and maintenance of large lipid membranes.^18^ In addition, Di Paola and colleagues^64^ suggested that demyelination is present in the premanifest HD brain, whereas both myelin breakdown and axonal damage are present in manifest HD. This proposal was based on the observation of decreased FA and increased RD in the isthmus of the CC of premanifest patients compared with age‐matched and sex‐matched controls in the absence of any changes in AD.^64^ On the other hand, they reported both increased RD and decreased AD in the CC of patients with manifest HD compared with matched controls. Similarly, Rosas and colleagues^9^ showed increased RD in the WM of patients with premanifest HD, which correlated with impaired performance on neuropsychological tests and was proposed to reflect early deficits in myelin. Moreover, they suggested that axonal pathology, as shown by changes in AD, is present only later in the disease course.

All of the aforementioned interpretations are built on the assumption of a direct correspondence between a specific microstructural property of WM and variations in DT‐MRI metrics. However, although DT‐MRI metrics are often regarded as probes of WM microstructure, these indexes do not tap specifically onto biological subcomponents of WM microstructure^65^ in that the tensor measured in the DT‐MRI model is an average of all the cellular compartments within a specific voxel.^66^ It is therefore very hard to interpret changes in DT‐MRI metrics in terms of changes in specific microstructural properties.^67^ Very different configurations of, for example, axonal packing, axonal size, and myelination may generate very similar outcome measures. Furthermore, RD and AD become difficult to interpret when multiple fiber orientations are present within a voxel, such as at fiber bundle crossings,^68^ and this situation affects between one and two thirds of the voxels in a human brain.^69^, ^70^

Specifically, these interpretations are based on the assumption that AD and RD are uniquely sensitive to axonal degeneration and demyelination, respectively.^71^, ^72^, ^73^, ^74^, ^75^, ^76^, ^77^ Such inferences are based on one study on Shiverer mice, where changes in myelin content were linked to increased RD, but unchanged AD.^73^ However, an increase in RD might not necessarily correspond to myelin loss; rather, it can have multiple meanings, including axonal loss.^67^ Furthermore, in regions of crossing fibers, increased RD within a specific tract could be attributed to a less coherent alignment of fibers, more crossing fibers from other bundles, lower density or less myelination of the fibers, or a combination of any or all of these factors.^67^, ^68^

Biophysical models of diffusion MRI, such as neurite orientation and dispersion density imaging^78^ and the composite hindered and restricted model of diffusion^66^ model compartment‐specific water diffusion to dissociate hindered extracellular and restricted intracellular diffusion properties of WM and should therefore provide a more biologically specific characterization of WM microstructural organization within the neural system.^79^

Zhang and colleagues^80^ used neurite orientation and dispersion density imaging to examine WM pathology in patients with premanifest HD. They reported widespread reductions in the neurite density index—a proxy of axonal density—in tracts including the CC and in WM surrounding the basal ganglia of patients with presymptomatic HD. Importantly, axonal density reductions in callosal regions predicted clinical markers of disease progression. Finally, increased coherence of axonal organization, as suggested by a smaller orientation dispersion index, was shown in patients with HD in tracts surrounding the basal ganglia and in the internal and external capsules, suggesting the presence of possible compensatory pruning of the axons in WM regions. Nevertheless, the estimation of specific tissue quantities from these models requires simplifying assumptions whose accuracy and generality, in the HD brain especially, are unknown. These techniques indeed still represent a relatively simple approach to modeling neural tissue and therefore cannot fully characterize pathological changes in WM microstructure.^81^

Quantitative magnetization transfer (qMT) imaging has enabled greater sensitivity to myelin content in WM.^82^ This method models the exchange rate between macromolecular protons and protons in surrounding tissue water when macromolecular protons are subjected to a radiofrequency pulse with a frequency that is off‐resonance for protons in free water.^83^ One of the outcome measures of qMT, the macromolecular proton fraction (MPF), has been shown to reflect demyelination in Shiverer mice and puppies,^82^, ^84^ to be sensitive to dysmyelination processes in multiple sclerosis patients,^85^ and to reflect myelin content of WM in postmortem studies of brains with multiple sclerosis.^86^ A study by Bourbon‐Teles and colleagues^63^ used diffusion tensor imaging and qMT to investigate HD‐related effects on WM pathways of the basal ganglia and motor systems. Specifically, the study compared patients with HD (24 manifest and 1 premanifest) to age‐matched and sex‐matched healthy controls. Although the patients with HD relative to controls exhibited significant reductions in an MPF component with high loadings of MPF in all WM regions, no differences were observed for components with loadings of AD, RD, or FA. The authors concluded that this pattern of results was consistent with a myelin impairment in HD. Interestingly, the MPF component score of the 1 premanifest individual in the study differed more than 3 times the standard deviation from the control mean. This observation suggests that MPF might already be reduced prior to disease onset and that MPF should be assessed as an early disease biomarker in a group of premanifest gene carriers.^63^

Although MPF is sensitive to WM myelin, this metric can also be affected by changes in cells and water content because of inflammation.^87^ Odrobina and colleagues,^88^ for example, measured MPF ex vivo in a demyelinated rat sciatic nerve and confirmed its correlation with myelin content, but also noted the difficulty of separating demyelination from inflammation by qMT alone. Nevertheless, although in manifest HD it is likely that inflammation goes hand in hand with myelin breakdown,^89^ a recent cerebrospinal fluid biomarker study found no evidence of neuroinflammation in early‐manifest HD.^90^ It is therefore plausible that changes in the MPF observed in this study were a reflection of aberrant myelination in these patients.

Human MRI studies have also shown that HD is associated with changes in iron levels across several brain areas.^4^, ^23^, ^29^, ^30^, ^91^ Tissue iron can be measured by MRI in vivo through its effect on transverse relaxation times (T2). Ferritin (the storage protein of iron) has been shown to strongly affect the MRI signal and markedly shorten T2 both in vitro and in vivo.^92^, ^93^

Increased ferritin levels are already present in the premanifest stage of HD.^91^, ^94^ As oligodendrocytes are the major iron‐containing cells in the adult central nervous system,^95^ the increased density of oligodendrocytes in HD that was demonstrated in mice models^22^, ^23^ should result in significant increases in iron and ferritin content in the HD brain. Evidence for increases in MRI‐based iron measures in HD further supports the suggestion for a homeostatic increase in oligodendrocytes as an active repair mechanism in patients with premanifest HD.

Early and heavily myelinated fibers are the most susceptible to myelin breakdown in HD,^4^ with WM degeneration starting in the caudate and putamen striatum structures and then spreading in a predictable, bilateral, and symmetric pattern to involve other earlier myelinating regions. In turn, later myelinating regions such as the medial temporal lobe are left much less affected.^29^, ^96^ This is consistent with neuroimaging evidence that the earliest WM changes in HD are seen before disease onset in early myelinating regions such as around the striatum, within the CC, and in posterior WM tracts.^12^, ^34^, ^36^, ^39^ The spatial pattern of pathology is in contrast with the “last‐in‐first‐out” hypothesis that was proposed for degenerative processes of normal brain aging,^97^ which postulates that later myelinating fibers are more vulnerable to insult in later life compared with earlier myelinating fibers.

Consistent with the suggestion of a dissociation between early and late myelinating regions in WM impairment in HD, Bartzokis and colleagues^4^ showed decreased ferritin iron levels in HD in the genu of the CC and frontal WM (late‐myelinating regions) and increased ferritin iron levels in the basal ganglia (an early myelinating region). Decreased iron levels in late‐myelinating regions are proposed to be attributed to suboptimal iron availability because of a redistribution of iron toward earlier myelinating regions. Furthermore, although remyelination processes may successfully compensate for myelin loss during the premanifest HD stage, these may start failing in later years, likely explaining evidence of decreases in iron content found between the patients with premanifest and symptomatic HD.^12^, ^91^

## Discussion and Future Directions

In the present review, we discussed evidence of WM abnormalities in HD. Although this disease has been tightly linked to striatal GM degeneration, an accumulating body of evidence suggests that alterations in WM microstructure are present early in HD progression,^1^, ^6^, ^17^, ^19^, ^53^ even in children at risk for HD,^18^, ^54^ and possibly even before any changes can be detected within the striatum.^19^ This implies that WM disturbances might independently contribute to HD pathogenesis^4^, ^17^, ^19^ and that, rather than being secondary to axonal insult in the form of Wallerian degeneration,^2^, ^80^ they might be a direct result of myelin and oligodendrocytes disturbance.^4^, ^14^, ^15^, ^20^, ^98^ Demyelination has been associated with severe disabilities in many developmental, psychiatric, and neurodegenerative diseases.^26^ Therefore, it is possible that also in HD, myelin loss, which leads to altered axonal conduction and axonal damage, may be directly responsible for some of the clinical symptoms. Furthermore, oligodendrocyte dysfunction early in the disease course may impair the processes of remyelination and myelin repair.^4^

A number of studies on HD animal models^22^, ^32^, ^51^, ^52^, ^99^ have lent support to the demyelination hypothesis of HD.^4^ Importantly, it is essential to consider these findings while always keeping in mind the equivalent and divergent features of the brain of different species. Nevertheless, studies on human HD carriers also suggest the presence of myelin dysfunction.^4^, ^9^, ^14^, ^63^, ^64^ However, most studies assessing WM changes in vivo have employed diffusion tensor imaging–MRI based metrics to quantify these alterations, even though these indexes do not tap specifically into biological subcomponents of WM microstructure.^65^, ^100^, ^101^ Recent advances in MRI techniques and tissue modeling enable a better characterization of the biological properties of WM microstructure and allow more specific monitoring of changes in these properties, both longitudinally and noninvasively.^102^ Longitudinal studies designed to relate specific microstructural changes to the genesis of pathology, while controlling for individual differences such as the impact of environmental factors, will prove particularly useful. Although it is evident that our understanding of the HD brain will increasingly rely on advanced MRI techniques, this reliance highlights the need to remember and address the shortcomings of these methodologies.

The identification of early changes in the brain WM microstructure in patients with HD is of fundamental importance as it allows insight into disease pathogenesis and progression and, further, might prove useful for the identification of disease‐related biomarkers and in measuring outcomes of clinical trials. In many neurodegenerative diseases such as Alzheimer’s and Parkinson’s diseases, myelin disturbance starts before other pathological changes are evident.^4^, ^103^ Similarly, in HD, critical pathogenic events might be present prior to neuronal death, and there might be a decades‐long period in which therapeutic intervention could change the course of the disease, before clinical evidence, such as behavioral, cognitive, and motor decrements, appear. Nonetheless, in vivo investigation of demyelination in patients with premanifest and symptomatic HD remains relatively unexplored when compared with other neurodegenerative diseases such as multiple sclerosis, and very little research has been carried out on the HD brain using these approaches.

To conclude, we reviewed the evidence that implicates WM aberrations and in particular impaired myelin and oligodendrocytes in HD pathology and suggests that these could constitute important targets for the study and early treatment of HD.

## Author Roles

(1) Research Project: A. Conception, B. Organization, C. Execution; (2) Manuscript: A. Writing of the First Draft, B. Review and Critique.

C.C.: 1B, 2A

A.R.: 2B

I.L.: 2B

D.J.: 1A, 2B

C.M.‐B.: 1A, 2B

## Financial disclosures of all authors (for the preceding 12 months)

C.C. reports employment with Cardiff University. A.R. reports employment with Cardiff University; advisory board of Roche; contracts with European Huntington's Disease Network (EHDN); and honoraria from California Institute for Regenerative Medicine (CIRM). A.R. also reports the following grants: May 2018–April 2021, Medical Research Council (MRC), UK Li, and Rosser, How CTIP2 Deficiency Drives Medium Spiny Neuron Degeneration and Dysfunction: Implications in Huntington’s Disease Pathogenesis; September 2017–February 2021, Health and Care Research Wales, RfPPB‐16a‐1298 (A.E. Rosser CI), Trial Designs for Delivery of Novel Therapies for Neurodegeneration (TRIDENT); June 2015–March 2021, National Institute for Social Care and Health Research Wales (W.P. Gray, A.E. Rosser, Y. Barde, M. Busse, V. Crunelli, S.B. Dunnett, R. Tudor Edwards, P. Eslambolchilar, K. Hamandi, K. Hood, D. Jones, M. Kerr, P. Morgan, M. Rees, N. Robertson, M. Wardle), Brain Repair And Intracranial Neurotherapeutics (B.R.A.I.N. unit) Wales; October 2018–September 2022, H2020‐MSCA‐ITN‐2018 (Marie Skłodowska‐Curie Innovative Training Networks), proposal number 813851, Training for Advanced Stem Cell Technologies in Neurology‐Advanced Stem Cell Technologies in Neurology (ASCTN) Training, Coordinator J. Canals (University Barcelona) Rosser and Allen at Cardiff University; January 2016–December 2019, Universistat Ulm, A.E. Rosser, Characterisation of the Natural History of HD and Assessment of Feasibility of HD Clinical Research Within the Network of HD Clinical Centres; Sep 2014 onward, Cure Huntington's Disease Initiative (CHDI), A.E. Rosser, A Prospective Registry Study in a Global Huntington's Disease Cohort (Enroll‐HD), Annual Renewal; and April 2017–July 2020, Campaign for Alzheimer’s Research in Europe (A.E. Rosser) Developing Stem Cell Technologies for the Neurodegeneration of Alzheimer's Disease. I.L. reports employment with the Max Planck Society. D.J. reports advisory boards of Connectom 2.0–Massachusetts General Hospital, Medical Research Council, and Centre and Unit Portfolio Review Board; and employment with Cardiff University, Australian Catholic University, and the Wellcome Trust. D.J. also reports the following grants: “Microstructural Imaging Data Centre (MIDaC)”; type: project grant; principal investigator: Griffin M.; co‐investigators: Murphy K., Charron C., Jones D.K., Hargrave P., Beltrachini L., Evans C.J., Papageorgiou A.; start date: October 2018; duration: 5 months; agency: Science and Technology Facilities Council (STFC); budget: £ 91,655; time per week = 0.4 hours. “Mapping Neurodevelopmental Trajectories for Adult Psychiatric Disorder: Avon Longitudinal Study of Parents and Children (ALSPAC)‐MRI‐II”; type: project grant; principal investigator: David A.S.; coinvestigators: Lewis G., Jones D.K., Zammit S., Bulmore E., Reichenberg A., Boyd A., Kempton M., de Stavolo B.; start date: October 2018; duration: 48 months; agency: MRC; budget: £ 2,202, 184; time per week = 1.88 hours. “Brain Repair and Intracranial Neurotherapeutics–the Wales BRAIN Unit–Renewal”; type: National Institute for Social Care and Health Research (NISCHR) unit; principal investigator: Gray W.G.; coinvestigators: Morgan P., Busse‐Morris M., Peall K., Li M., Rosser A., Barde Y., Crunelli V., Jones D.K., Lane E; Agency: NISCHR; budget: £800,000; time per week = 1 hour. “Characterising Brain Network Differences During Scene Perception and Memory in APOE‐e4 Carriers: Multi‐Modal Imaging in ALSPAC”; type: project grant; principal investigator: Graham K.S.; coinvestigators: Lawrence A.D., Jones D.K., Wise R.G., Kordas K., Zhang J., Mackay C.M., Filippini N., Saksida L.M.; start date: October 2016; duration: 48 months; agency: MRC; budget: £1,756,395; time per week = 2 hours. “The UK7T Network: Developing the Ultra‐High Field MRI Platform for Biomedical Research”; type: research grant; principal investigator: Bowtell R.; coinvestigators: Miller K., Carpenter T., Rowe J., Williams G., Wise R.G., Jones D.K., Linden D., Muir K., Goense J., Muckli L., Francis S., Glover P., Gowland P., Morris P., Bajaj N., Clare S., Jezzard P; start date: January 2016; duration: 36 months; agency: MRC; budget: £1,309,733; time per week = 0 hours. “Expansion and Relocation of CUBRIC’”; type: structure funds; principal investigator: Jones D.K.; start date: January 2015; duration: 72 months; agency: Welsh European Funding Office; budget: £4,578,475; time per week = 0 hours. *“*Computational Modelling and Prediction of Brain Shift to Improve Surgical Navigation”; type: industrial Cooperative Awards in Science & Technology (CASE) studentship; start date: January 2015; duration: 36 months; agency: Engineering and Physical Sciences Research Council (EPSRC)/Renishaw; budget: £53,78; time per week =1 hour. C.M.‐B. reports Bristol Research into Alzheimers and Care of the elderly (BRACE) Alzheimer's Charity Scientific advisory board, employment with Cardiff University, and the following grant: Duckers J et al, Cystic Fibrosis Memory Assessment and MRI Screening, Health and Care Research Wales,£19,200 for 12 months (start April 1, 2020).
